# Unmet need for autism-aware care for gynaecological, menstrual and sexual wellbeing

**DOI:** 10.1177/13623613241290628

**Published:** 2024-10-15

**Authors:** Richard O de Visser, Rachel Mosely, Julie Gamble-Turner, Laura Hull, Felicity Sedgewick, Charlotte Featherstone, Chella Quint OBE, Eloise Freeman, Marianna Karavidas

**Affiliations:** 1 Brighton and Sussex Medical School, UK; 2 Bournemouth University, UK; 3 University of Bristol, UK; 4 Autistica, UK; 5 Period Positive, UK; 6 Sheffield Hallam University, UK

**Keywords:** autism, health care, menopause, menstruation, sexual health

## Abstract

Autism can make menstruation and menopause and other aspects of reproductive and sexual health (RSH) more difficult. However, healthcare professionals (HCPs) often fail to provide autism-aware care, and often lack the skills or confidence to discuss RSH with patients. This study explored whether autistic people experience particular difficulties and have unmet needs when seeking RSH care. Online surveys consisting of forced-choice items and free-text boxes were completed by 136 autistic adults in the United Kingdom. Quantitative data were analysed using standard parametric and non-parametric tests. Qualitative data underwent thematic analysis. The data provide a clear picture of unmet needs for autism-aware healthcare for RSH. The quantitative data and qualitative analysis revealed that respondents felt: (a) uncomfortable discussing menstrual issues, menopause and sexual wellbeing; (b) that HCPs rarely accommodate their sensory needs and communication preferences; and (c) that HCPs rarely demonstrated awareness of how autism can affect RSH. This novel study highlighted widespread unmet needs for appropriate RSH care for autistic people. Effort is required to enhance HCPs’ ability to provide autism-aware care for RSH. The findings could inform the development of resources and training to improve healthcare for autistic people.

**Lay abstract:**

Autistic people often experience difficulties with healthcare, and are more likely than neurotypical people to have unmet healthcare needs. They may also be more likely to find menstruation and menopause more difficult than neurotypical women. Healthcare professionals (HCPs) often have insufficient training and support to work with autistic adults, and they often lack the skills or confidence to discuss reproductive and sexual health (RSH) with patients. When these two issues are combined, it would appear that autistic people may experience particular difficulties when seeking RSH care. The aim of this study was to explore autistic people’s experiences of healthcare related to RSH in the United Kingdom. Surveys were distributed with assistance of an autism charity, and were completed by 136 adults. The survey consisted mainly of tick-box responses, but there were also several opportunities for participants to write comments about their experiences. Respondents felt that HCPs almost never seem to know how autism affects their RSH. There was broad agreement that HCPs need to be more aware of the impact of autism on healthcare experiences in general, and the specific impacts of autism on RSH. The data provide a clear picture of unmet needs for autism-aware healthcare for RSH, but further research is required to explore HCPs’ knowledge about how autism affects RSH. Combined with our findings, such research could inform the development of resources and training to improve healthcare for autistic people.

Autism is a neurodevelopmental condition and a biopsychosocial phenomenon: heightened sensory sensitivity influences psychological states and behaviours, which affect social interactions (American Psychiatric Association, 2013; Schauder & Bennetto, 2016). The work autistic people do to manage social interactions and ‘camouflage’ or ‘mask’ autistic traits may contribute to poorer physical and psychological wellbeing (Lai et al., 2019).

Autistic people are more likely than neurotypical people to report unmet healthcare needs (Black et al., 2022; Doherty et al., 2022; Mason et al., 2019). Physical features of healthcare settings – for example, bright lighting, harsh unpredictable noises, clinical furnishings and unpredictable waiting times in busy shared-spaces – may present particular challenges (Black et al., 2022; Mason et al., 2019). Important interpersonal barriers include healthcare professionals’ (HCPs’) lack of awareness of, or unwillingness to adapt to, autistic people’s needs (Mason et al., 2019). Autistic people value being listened to, and attended to holistically, but this may be difficult given existing demands and resource constraints within primary care, and insufficient training and support for HCPs to work with autistic adults (Corden et al., 2022; Malik-Soni et al., 2022).

## Menstruation, menopause and sexual wellbeing

Differences in sensory sensitivity and social interaction may make the physical and psychosocial changes of menstruation and menopause more challenging for autistic people assigned female at birth (AFAB) than their neurotypical peers (Groenman et al., 2022; Karavidas & de Visser, 2022; Moseley et al., 2020a, 2020b). Like autism, menstruation and menopause are biopsychosocial phenomena. Just as autistic people report needing to mask or camouflage their autism, many women report having to manage or mask symptoms of menstruation and menopause in public contexts (Butler, 2020; Cronin et al., 2024). Autistic characteristics such as sensory sensitivity and social interactional differences may be heightened during menstruation, and autistic people may find it more difficult to express their needs and to receive support (Gray & Durand, 2023; Steward et al., 2018).

There are intersections between autism and broader sexual wellbeing. Autistic people report poorer RSH education (Graham Holmes et al., 2022). They are more likely to report adverse sexual experiences (Bargiela et al., 2016; Gibbs et al., 2021), and to reject gender binaries or identify with a gender different from that assigned at birth (Kallitsounaki & Williams, 2023). However, general practitioners (GPs) often lack the skills or confidence to discuss RSH, meaning that RSH needs may not be properly addressed (Dintakurti et al., 2022; Dyer & das Nair, 2013; Kelder et al., 2022). It would appear, therefore, that autistic people may experience particular difficulties in primary care consultations for RSH care. However, there is a need for better understanding of the reproductive and sexual health (RSH) care needs and experiences of autistic people.

## Study aims

We aimed to explore autistic people’s experiences of RSH in the context of primary care, because in the United Kingdom most healthcare for these issues is provided by GPs in the first instance. We used a mixed-method online questionnaire to explore experiences of RSH care, and how these relate to other healthcare experiences and to personal characteristics. The study was designed to inform subsequent studies of HCPs’ knowledge, beliefs, competencies and behaviours. The long-term aim is to develop resources to improve HCPs’ capacity to address the RSH needs of autistic patients.

## Materials and methods

### Sample

The questionnaire was completed by 136 autistic people AFAB aged 18 to 71 years. The ages at which respondents were diagnosed with, or identified as autistic, ranged from 2 to 66 years. Years since autism diagnosis/identification ranged from 0 to 48 years. Table 1 presents descriptive demographic data. Compared to the general population, there were more white people and people with higher levels of education.

**Table 1.** Description of sample (*n* = 136).

Age (years) range	18–71
mean (SD)	40.2 (12.2)
median	40
Age at autism diagnosis range	2–66
mean (SD)	35.3 (13.4)
median	37
Years since diagnosis range	0–48
mean (SD)	5.2 (7.7)
median	2
Gender	**Proportion**
Female	92%
Non-binary	4%
Other	4%
Ethnicity	
White	88%
Asian/Asian British	2%
Black/Black British	2%
Middle Eastern	2%
Jewish	2%
Hispanic	2%
Mixed/dual heritage	2%
Highest completed level of education	
Higher degree (e.g. MA, PhD)	42%
Undergraduate degree (e.g. BSc)	36%
Secondary school	17%
Did not complete secondary school	5%
Autism diagnosis?	
Formally diagnosed	72%
Seeking formal diagnosis	12%
Self-diagnosed	16%
Other condition?	
Neuro-developmental condition	34%
Mental health condition	79%
Mobility-limiting condition	34%
Menstrual/menopausal status	
Pre-menopausal	54%
Peri-menopausal	25%
Post-menopausal	19%
Not sure	2%

Many respondents reported additional neurodevelopmental conditions – most commonly attention-deficit/hyperactivity disorder (21% of sample), dyslexia (10%) and dyspraxia (7%). Most respondents reported a psychological condition, with 64% reporting an anxiety-related disorder and 48% reporting depression. Many respondents reported a condition that impaired their mobility, the most common being hypermobility (13% of sample), fibromyalgia or other chronic pain (10%), and Myalgic Encephalitis/Chronic Fatigue Syndrome (4%). Less than one-quarter described their physical health as ‘very good’ (21%) or excellent (2%). Only 10% described their psychological wellbeing as ‘very good’, and none labelled it ‘excellent’.

Reported age at menarche ranged from 9 to 16 years (M = 12.5, SD = 1.7, median = 13). Among pre-menopausal respondents, 71% reported regular periods. Ratings of menstrual pain averaged 5/10 (M = 5.0 (SD = 2.7), median = 5), but 24% gave ratings of at least 8/10.

### Measures

The exploratory nature of the study and the lack of relevant survey questions demanded the creation of many items (supplementary file).

#### Neurodivergence, mental health and other conditions

Respondents indicated whether they had been formally diagnosed as autistic, and their age at diagnosis or when they first self-diagnosed or identified as autistic. Respondents also indicated any other neuro-developmental conditions they had been diagnosed with: first they indicated whether they had such any such condition, and then they used a free-text box to describe the condition(s). Similar questions were asked about mental health conditions, and conditions that affect mobility. Respondents used a 5-point scale (*Excellent – Very good – Good – Fair – Poor*) to rate their overall physical and psychological wellbeing.

#### Menstrual history

Respondents indicated how old they were when they had their first menstrual period, and whether they had any experience of the menopausal transition (categorised as *No – perimenopause – post-menopause – don’t know*). They also indicated whether their periods are or were regular, and used a 10-point scale (*not at all – extremely*) to indicate how painful their periods are or were. Other studies of menstrual pain have found that numeric scales provide comparable data to other response formats, but are easier and more convenient to use (e.g. Larroy, 2002).

#### Discussion of health concerns

Respondents used a 10-point scale (*not at all – extremely*) to indicate how comfortable they felt discussing six specific topics with HCPs in primary care: physical health, psychological wellbeing, sexual health, menstrual-related issues, menopause-related issues and autism. They used the same 10-point scale to indicate how comfortable they would be discussing each of these topics (excluding autism) in a primary care context via four different modes: in person, video call, email, phone call. They then indicated their preferred mode of discussion for each topic. They also indicated whom they would prefer to initiate conversations about each of these topics (*me* – *doctor* – *no preference* – *I would not want to discuss this*). Participants could add free-text comments.

#### HCPs’ communication styles

The final section asked respondents how often (never/sometimes/usually/always) HCPs in general practice engage in specific behaviours: Explain things in a way that is easy to understand; Give you the opportunity to ask questions; Check that you understand what they say; Give you enough time to process what they say; Check that they are communicating with you in your preferred way; and Accommodate your sensory needs. Participants could also enter free-text comments. The same scale was used to assess how often HCPs were perceived to understand how autism affected the respondent’s sexual health, experiences of menstruation, and (if applicable), experience of menopause. They could also enter free-text comments.

### Procedure

Four autistic members of the research insight group of the UK charity Autistica gave feedback on a draft of the questionnaire, and were reimbursed according to best practice guidance (NICE, 2022). Following Institutional Review Board authorisation and approval from Autistica, the questionnaire was distributed by Autistica via its email list. Self-identified or formally diagnosed autistic people AFAB were invited to take part by following a link to the online survey. Only adults with capacity to give individual informed consent were eligible to participate. The survey link took potential participants to an information sheet and consent form. All who completed the questionnaire were able to enter a draw for a £50 online shopping voucher. The Autistica mailing list contains of 13,000 contacts: it includes autistic people of all genders as well as non-autistic family members, researchers, and other professionals and non-professionals who are interested in issues that affect autistic people. Because it is not known how many people on the mailing list are autistic women, a response rate could not be calculated.

### Analysis

In addition to descriptive statistics, we used multivariate analysis of variance (MANOVA) to explore whether responses varied with additional neuro-developmental conditions, psychological conditions, conditions that affected mobility, and recency of diagnosis. To reduce inflating the Type I error rate from multiple comparisons, we set the significance level at *p* < 0.01.

We analysed responses to open-ended questions using Thematic Analysis (Braun & Clarke, 2006). This entailed: (1) data familiarisation through reading all responses to each of the open-ended questions; (2) initial coding of responses to note key experiential content; (3) exploring initial codes to derive themes and subthemes; (4) further reviewing and refining themes to ensure that they accurately reflected the data; (5) finalising themes and exploring systematic variation according to time since diagnosis, menopause experience, and so on; and (6) reporting the findings. The analysis was conducted by R.O.dV. and E.F., who independently coded the free-text comments, and then compared codes and notes and engaged in discussion to derive an initial list of themes, which was then presented to all authors for consideration and confirmation. Each quote in the results section includes the respondent’s age and menopausal stage. Qualitative findings are integrated with the quantitative analyses, and the six themes are presented in three clusters. Themes 1a, 1b and 1c relate to the open-ended question about preferred communication mode. Theme 2 relates to the free-text question about HCPs’ autism-aware behaviours. Themes 3a and 3b relate to the open-ended questions about HCPs’ awareness of how autism affects RSH.

### Community involvement

The research insight group of the charity Autistica contributed to survey design, and recruitment was conducted via Autistica’s community networks.

## Results

### Comfort discussing RSH

Table 2 shows that respondents’ levels of comfort discussing various topics tended to be around the midpoint of the 10-point scale. Participants were significantly more comfortable discussing physical health than any of psychological wellbeing, menstrual-related issues, menopause-related issues, or sexual health (*F*(5, 615) = 27.24, *p* < 0.01). There were no significant differences in comfort levels for discussing psychological wellbeing, menstrual-related issues, menopause-related issues, or sexual health. However, the standard deviations indicated wide variability in comfort. For reference, participants also tended not to feel confident discussing autism in primary care (M = 4.83, SD = 3.19). Notably, greater confidence discussing autism was significantly correlated with greater confidence discussing menstruation, menopause, and sexual wellbeing (*p*s all <0.01).

**Table 2.** Comfort discussing health topics, preferred consultation more, and preferred initiator of discussions (*n* = 136: mean (SD)).

Health topic	Physical health	Psychological well-being	Menstrual-related	Menopause-related	Sexual Health
Comfort discussing topic	7.04 (2.51)	5.41 (2.89)	5.61 (3.05)	5.52 (3.23)	4.61 (3.12)
Time since autism diagnosis/identification					
⩽2 years	7.30 (2.57)	5.63 (2.92)	6.13 (3.05)	6.00 (3.27)	5.00 (3.04)
>2 years	6.75 (2.44)	5.16 (2.87)	5.04 (2.99)	4.98 (3.13)	4.18 (3.17)
Other condition?					
Neuro-developmental	7.47 (2.39)	6.18 (2.97)	6.21 (3.08)	6.13 (2.97)	5.37 (2.96)
Mental health	7.01 (2.61)	5.59 (2.89)	5.56 (2.95)	5.51 (3.11)	4.65 (3.13)
Mobility-limiting	7.24 (2.50)	5.22 (2.89)	5.93 (3.00)	6.00 (3.05)	5.15 (3.14)
Preferred consultation mode					
In-person	60.8%	60.3%	49.6%	57.9%	70.1%
email	26.4%	26.2%	37.6%	25.4%	18.9%
Phone	7.2%	7.9%	6.4%	7.1%	7.1%
Video	5.6%	5.6%	6.4%	9.5%	3.9%
Preferred initiator of discussion					
Self	39.3%	35.6%	41.0%	29.6%	37.3%
No preference	37.8%	29.6%	33.6%	38.5%	22.4%
Doctor	23.0%	31.9%	23.1%	28.9%	23.9%
Would not want to discuss this	0.0%	3.0%	2.2%	3.0%	16.4%

There were no significant correlations between current age and comfort discussing the six topics (*p*s all ⩾0.47). Years since diagnosis/identification were not significantly correlated with levels of comfort discussing all six topics (*p*s all ⩾0.05). Use of a two-category median-split variable indicated that people diagnosed within the last 2 years were similar to those diagnosed more than 2 years ago in levels of comfort discussing all six topics (*p*s all ⩾0.05).

Comfort discussing any of the topics did not significantly differ according to whether participants had another neuro-developmental condition (*p*s all ⩾0.06), a mental health condition (*p*s all ⩾0.56), or a condition that affects mobility (*p*s all ⩾0.34). Furthermore, there were no significant interaction effects between comfort levels and combinations of neuro-developmental, psychological, and mobility conditions (*p*s all ⩾0.40). In other words, the presence of other conditions was not related to people feeling less or more comfortable discussing these issues.

### Preferred communication mode for RSH

The middle section of Table 2 shows that for all topics, the preferred consultation mode was in-person, although this was less preferred for menstrual issues than for other topics. The second most popular mode was email, the mode least similar to in-person consultations given its lack of in-person contact, and the likelihood of not being conducted in real time. The qualitative data below provide possible explanations for this bimodal difference.

Preferred mode of communication about any of the five topics was not significantly associated with recency of diagnosis using a median-split variable (*p*s all ⩾0.27), nor whether people had another neuro-developmental condition (*p*s all ⩾0.23), a mental health condition (*p*s all ⩾0.78), or a condition that affected their mobility (*p*s all ⩾0.09)

#### Qualitative Theme 1a: benefits and drawbacks of in-person consultations

Analysis of the qualitative data helped to explain the data in Table 2: it revealed various positive and negative aspects of the different communication modes, and that each mode had benefits and drawbacks, depending on the individual and the topic of discussion. Important influences here included whether there was a need for a physical examination, and whether discussion would be emotionally uncomfortable in-person. Positive comments about remote consultations were often made in relation to sensitive or potentially embarrassing topics such as mental health, menstrual health, and sexual wellbeing:

For an injury it might be easier to show professionals in person. For something routine like contraceptive renewal or something I find harder to talk about (like mental health), email might be better. (29, pre-menopausal)

Commonly-reported positive aspects of in-person consultations included the perception that the physical proximity of in-person consultations allows people to better express their needs, to ensure that those needs are understood, and to ask questions:

In person helps me with central cohesion. I can understand the ‘bigger picture’. I can see how people respond and their body language helps me with communication. I don’t like video calls as much as I prefer to be in close proximity to the person I’m talking to. For me it feels like there’s a detachment from the situation. (62, post-menopausal)

Furthermore, some respondents preferred in-person consultations because they felt that the practice setting provides a space that is physically and temporally separate from everyday life, and which also ensures confidentiality that may not always be available in remote consultations:

It’s quiet and restful in there and private. I cannot be overheard and the walk and waiting are helpful transitions. [. . . I cannot be] overheard by the flats surrounding me or anyone on the street. (45, peri-menopausal)

However, many respondents identified negative aspects of in-person consultations. For example, some found the demands of in-person consultations too great, and felt more comfortable and confident in remote consultations of various forms:

If I don’t have the pressure of maintaining a demeanour including eye contact I am more comfortable and natural so I prefer telephone as the conversation tend to flow naturally. (31, pre-menopausal)

#### Qualitative Theme 1b: distance and connectedness

Many respondents made positive comments about video consultations, noting that they provide the visual element of in-person consultations while also providing the physical and psychological distance afforded by all remote consultations:

Video consults means I can be in the comfort of my own space, but still see the doctor and they can see me. we can pick up on each other’s non-verbal cues, which I find helpful when I am struggling to find the right words. (27, pre-menopausal)

However, some preferred to keep medical consultations separate from their home life:

I don’t like video because then my safe space at home gets ‘contaminated’ (not sure what word to use) by an appointment. I need to feel like I’m getting out of my house to go to an appointment. (27, pre-menopausal)

#### Qualitative Theme 1c: concerns about control and security

Some respondents appreciated the asynchronous nature of email exchanges because this reduced the pressure of having to engage in real time and afforded a greater sense of control within the interaction:

I find it much easier to express myself in writing and to process written communication. It allows me to time to think what I want to see. It also means sensory sensitivities are avoided and it can be a planned interaction. (43, pre-menopausal)

Whereas some participants appreciated that emails left a record of communication that could be referred to and checked if necessary – for example, ‘there is a trail of what is being said’ (20, pre-menopausal), others were concerned about data security – for example, ‘what if my emails get hacked?’ (pre-menopausal).

### Who should initiate communication about RSH?

The lower section of Table 2 shows wide variation in who participants would prefer to initiate discussion of each topic in primary care consultations. Several points are noteworthy. First, for no topic did a majority identify any single option. Second, when people expressed a preference, it was that they – rather than their GP – should raise the issue. However, around one-quarter wanted their GP to raise these issues. Importantly, one-sixth of respondents reported that they would not want to discuss their sexual health with their GP.

Using the median-split variable, recency of diagnosis was not significantly associated with who respondents preferred to initiate discussion of each of the five topics (*p*s all ⩾0.29).

For menstruation-related issues, respondents who had a mental health condition were significantly more likely to prefer that their doctor initiated discussions and significantly less likely to prefer to raise the issue themselves (*p* < 0.01). Otherwise, there were no significant associations between who participants would prefer to initiate discussion of each topic and whether they had another neuro-developmental condition (*p*s all ⩾0.14), a mental health condition (*p*s all ⩾0.01), or a condition that affected their mobility (*p*s all ⩾0.02).

### HCPs’ autism-aware behaviours

Table 3 reports how often doctors or nurses enact various patient-friendly behaviours. The first three are not autism-specific, but are relevant and important for all patients. Less than one-fifth of respondents reported that HCPs always explain things in a way that is easy to understand, always give them the opportunity to ask questions, or always check that what they say has been understood. The final three behaviours are autism-specific: HCPs tend not to engage in behaviours that would help autistic people to be actively involved in their health care. Nearly half of the respondents reported that HCPs never give enough time to process what they have said, and over two-thirds reported that HCPs never check that they are communicating in their preferred mode (e.g. in-person or online), and never accommodate their sensory needs.

**Table 3.** Perceptions of prevalence of autism-aware practices by healthcare professional in primary care contexts (*n* = 136).

	Never	Sometimes	Usually	Always
**How often do primary care staff . . .**				
Explain things in a way that is easy to understand?	3.2%	34.1%	46.8%	15.9%
Give you the opportunity to ask questions?	8.7%	32.5%	38.9%	19.8%
Check that you understand what they say?	32.5%	46.0%	15.9%	5.6%
Give you enough time to process what they say?	41.9%	37.9%	16.1%	4.0%
Check that they are communicating in your preferred way?	71.8%	20.2%	6.5%	1.6%
Accommodate your sensory needs?	69.8%	20.8%	6.6%	2.8%
**How often do primary care staff seem to know how your autism affects . . .**				
. . . your sexual health	92.2%	4.9%	1.9%	1.0%
. . . your experience of menstruation	93.3%	2.9%	1.9%	1.9%
. . . your experience of menopause (if applicable)	95.8%	2.1%	2.1%	0.0%

Whether respondents felt that HCPs explain things in ways that are easy to understand, allow them to ask questions, check that they understand information, give time to process information, check for their preferred communication mode, or accommodate their sensory needs was not significantly associated with recency of diagnosis (*p*s all ⩾0.05), nor with the presence of other neuro-developmental conditions (*p*s all ⩾0.15), mental health conditions (*p*s all ⩾0.03), or conditions that affect mobility (*p*s all ⩾0.26).

#### Qualitative Theme 2: sensory and processing allowances

Free-text responses indicated that some issues related to sensory sensitivity were applicable to a range of healthcare settings and interactions. These included sensory responses to aspects of the physical environment:

It’s hard to accommodate sensory needs in the type of buildings that practices often are. The light and the noise can be hard to control. That’s why remote options help me, I can be in my own safe environment and focus on the conversation. (pre-menopausal)

Added to these general challenges were the specific challenges of gynaecological concerns and their diagnosis and treatment. However, some participants also noted that they were rarely asked about their specific needs or encouraged to express these, and others noted that attempts to have their needs met were not always responded to appropriately:

I need encouragement and support to be able to ask questions in a healthcare setting, and because I don’t receive this, I often don’t ask questions that I would like to. (52, peri-menopausal)

They never ask anything about sensory or neurodiversity needs. Although they listen, I don’t think they understand from your point of view and are quick to dismiss things, 5 years I’ve been fighting to have some sort of action regarding heavy periods being sent away, only recently speaking to a female ‘womans’_[sic]_ champion. (44, peri-menopausal)

Perhaps not surprisingly given differences in sensory sensitivity, many respondents found gynaecological examinations particularly difficult, but reported an absence of appropriate responses to their specific needs:

The attempted [cervical] smear experience left me traumatised and in pain for over a week. For several years I feel my period issues were ignored and dismissed because I’m autistic and I wasn’t believed and taken seriously. (35, pre-menopausal)

### HCPs’ awareness of how autism affects RSH

The bottom section of Table 3 shows that respondents overwhelming reported that HCPs were not aware of the impact of autism on aspects of RSH: very few reported that HCPs ever seem to know how their autism affects their sexual health, their experiences of menstruation, or (if relevant) their experiences of menopause. There was too little variation in responses for analyses of variation by time since diagnosis, or presence of other conditions.

Analysis of free-text responses indicated two broad themes outlined below. The first included responses from people whose autism was not known about by HCPs or those who did not raise the issues of autism and how it affects their RSH. The second focused on experiences of people who felt that HCPs did not act appropriately despite having some awareness of how autism might affect RSH.

#### Qualitative Theme 3a: lack of awareness of autism and/or its effects

Some respondents reported that HCPs were unaware that they were autistic. In some cases, this reflected the absence of a formal diagnosis; in others, respondents had not disclosed their autism diagnosis to primary care staff. For other respondents, autism was not a part of discussions about RSH because respondents were not sure whether or how autism affected this domain of their wellbeing:

I have never been asked about my autism in the context of the above. I haven’t raised it, but I wouldn’t know how to introduce it into the conversation, and to be honest also don’t really know myself how my autism affects these things (I’m quite newly diagnosed). (46, peri-menopausal)

However, in other cases, respondents deliberately withheld their autistic status. In some cases this was because they did not feel that this would lead to any changes in care:

They don’t know I am [autistic]. I have carefully kept neurodivergence and all my mental health stuff off my medical records, and I censor what I say, and mask heavily so it won’t be picked up on. (45, peri-menopausal)

Among respondents whose autism was known by primary care staff, the predominant experience was that it was not deemed a potential influence on their sexual wellbeing, or their experiences of menstruation or menopause. Some participants noted that normative assumptions pervaded all health care, and that apparent ‘normality’ in consultations masks the potential impact of autism:

It is not a consideration at all – I definitely feel I am treated as if I am neurotypical, although it could be because I present as very high functioning. (33, pre-menopausal)

#### Qualitative Theme 3b: awareness not accompanied by appropriate action

Whereas responses in the previous theme related to HCPs’ lack of awareness of autism, many other respondents felt that HCPs who knew about their autism were either unaware of its potential effects, or actively downplayed or dismissed the influence of autism on RSH:

I’ve tried to explain that autism affects my menstrual cycle and also how I respond to medications and treatments and that I’m wary of side effects and symptoms as I can be highly sensitive to these things, but I’ve been told it’s nonsense or been given bemused looks. (43, peri-menopausal)

Some participants noted that their past experiences of healthcare made them uncertain about discussing sexual health or reluctant to do so. Their comments suggested that HCPs’ lack of appropriate responses to discussions of the impact of autism on more general physical health was likely to extend to the specific domain of RSH:

I haven’t discussed menstruation and sexual health with my doctors since I was diagnosed with Autism but I have been to discuss a physical health condition and the doctor didn’t want to hear about how the Autism might make me have different needs. (45, pre-menopausal)

Others noted that when they expressed interest in learning how to manage the effects of autism on their RSH, HCPs tended to respond inappropriately, indifferently, or in ways that suggested a lack of knowledge or expertise. The second quote below indicates that on the rare occasions that HCPs had good intentions, they often lacked the capacity to respond appropriately:

I recently tried to talk to my GP about menopause but was told to read up on the internet and make a decision on what I wanted. (50, peri-menopausal)

The nurse was really nice about my finding appointments difficult, and said her son had social anxiety, so she understood it could be hard. However, there was no indication that she understood e.g. that being touched in order to administer certain contraceptives would be an issue. (28, pre-menopausal)

In some free-text responses, participants noted that even when autism and its effects on RSH were acknowledged, this did not always lead to better experiences or outcomes, because there was a lack of effective actions or treatments:

There is some recognition of possible differences compared with neurotypical patients, but not what to do about them/lack of clear options for obtaining solutions. (54, post-menopausal)

Other respondents noted an unevenness in awareness and appropriate behaviour among HCPs. For example, whereas some HCPs tried to understand and respond to the impact of autism on gynaecological health, many others were unaware, or did not act appropriately on the awareness that they did have:

My GP has tried to be understanding about how autism affects my menstrual cycle but other professionals she referred me to didn’t understand at all and it took a long time to find a solution. (19, pre-menopausal)

It would have been helpful if they’d considered sensory aspects to my periods and the pill before putting me on it and when discussing it with me. (22, pre-menopausal)

## Discussion

This exploratory study addressed autistic people’s experiences of healthcare related to menstruation, menopause, and broader sexual wellbeing. The combination of fixed-response and free-text questions provided evidence that many of the unmet needs identified in studies of health in general appear to be accentuated in the specific context of RSH (Black et al., 2022; Corden et al., 2022; Doherty et al., 2022; Malik-Soni et al., 2022; Mason et al., 2019).

In relation to menstrual wellbeing, many respondents reported irregular periods and/or high levels of period pain (Gray & Durand, 2023; Steward et al., 2018). However, respondents tended not to feel comfortable discussing gynaecological and sexual wellbeing, despite many also expressing a desire to initiate discussion of these issues. It is important for HCPs to be aware of these somewhat paradoxical observations, and to be prepared to work with autistic people – as individuals and as a community – to facilitate discussion of these important issues.

Comfort discussing RSH was not related to how long it was since people became aware of their autism, or whether they had neuro-developmental, mental health, or physical health conditions: levels of comfort were quite low for all participants, and did not differ significantly between groups. Similarly, reports of whether HCPs enact autism-friendly practices indicated that it is not only specific subgroups, but all autistic people who reported a low prevalence of appropriate behaviours. The significant correlations between less comfort discussing gynaecological issues and less comfort discussing autism may be due to both domains carrying stigma.

There was no consensus about the communication mode that was most preferred or considered most appropriate for RSH. Indeed, the qualitative data revealed that preferred communication mode was also affected by the nature of the health concern: some preferred remote consultations for potentially embarrassing topics, but it was also noted that in-person consultation were preferable for discussing concerns about psychological issues or psychological aspects of physical health concerns. This has been noted in studies of remote consultations in the general population (de Visser et al., in press), and many of the comments about healthcare in general corroborate the findings of previous studies of autistic people (Black et al., 2022; Corden et al., 2022; Doherty et al., 2022; Malik-Soni et al., 2022; Mason et al., 2019).

A strikingly clear novel finding was that respondents did not feel that HCPs behaved in ways that indicated an awareness of how autism may affect RSH. This appeared to be an intensification of a broader absence of autism-aware healthcare (Corden et al., 2022; Doherty et al., 2022; Malik-Soni et al., 2022).

Many respondents reported that they had deliberately withheld information about their autism, because they found it easier to accommodate their own autistic needs than revealing personal information that may not be responded to appropriately. This suspicion was confirmed in the reports of participants who had disclosed their autistic status: they had experiences of HCPs ignoring the potential impact of autism on their sexual wellbeing, denying that it could have an effect, or not responding in ways that indicated that they possessed the required knowledge, skills, or motivation. These experiences corroborate literature around the particular healthcare difficulties of autistic people (Gosling et al., 2023; Grove et al., 2023).

When combined, the quantitative and qualitative data provide a clear picture of unmet needs accompanied by the message that there is no one-size-fits-all approach to improving healthcare for RSH among autistic people. This raises the question of how to facilitate tailoring of communication methods to match patients’ general preferences and their specific wishes related to specific topics. One approach may be to ask patients for their preferences at the time of arranging each appointment (Nicolaidis et al., 2016). This could prompt and help HCPs to adopt modes of communication that are sensitive to, and responsive to, the needs of autistic people.

The data relating to whom participants preferred to initiate discussions of different topics also revealed a lack of consensus and considerable variation between topics. This suggests a need to identify how to allow and encourage autistic people to indicate their preferences. One option could be a checklist or pro forma to be completed prior to a consultation. Recent research has explored the potential use of Autism Health Passports: paper-based or digital tools that patients can use to describe their accessibility needs (Ellis et al., 2023). Such mechanisms are recommended in UK clinical guidance, because they may help to address misunderstandings between patients and HCPs, promote person-centred care, and improve patient safety (NICE, 2012; Northway et al., 2017). However, more evidence is needed about their effectiveness and how to improve their acceptability and impact (Ellis et al., 2023).

### Strengths and limitations

This was the first known study to focus on autistic people’s experiences of discussing RSH in UK primary care settings. A strength was the use of both closed and open questions to allow for comparison between individuals with potentially relevant co-occurring conditions, as well as additional rich qualitative data. Autistic adults often have complex profiles of physical and mental health conditions (Lai, 2023; Ward et al., 2023). However, we found little evidence that co-occurring conditions affected healthcare experiences. It must be noted that because the study did not include a comparison group of non-autistic people, we cannot determine whether the experiences described here are unique to autistic people, or reflect more general experiences of RSH.

Our sample may not be representative of the autistic population as a whole: Black and ethnic minority autistic people were underrepresented, as were non-binary and transgender individuals to whom these issues are also pertinent. Although the reported experiences with HCP might be common to autistic people, they may not capture intersectional difficulties – for example, being autistic and transgender in RSH contexts (Wallisch et al., 2023). Recruitment through social media often results in more highly educated and later-diagnosed samples (Rødgaard et al., 2022), and it may have affected the proportion of respondents who preferred remote and/or asynchronous communication modes. This may limit the ability to generalise the findings. Furthermore, the study design precluded the involvement of autistic people who were unable to access the survey or give individual informed consent, such as those with profound intellectual disabilities. Furthermore, the sample was self-selected: the topic may have been most salient to those with more problematic sexual and menstrual health and/or health care.

## Conclusion

Quantitative and qualitative data highlighted unmet needs for autism-aware care for RSH. There was broad agreement that HCPs need to be more aware of the impact of autism on healthcare experiences in general, and in the specific domains of menstruation, menopause, and sexual wellbeing. Furthermore, they need to address these topics in ways that acknowledge the general needs of each patient, and their specific needs across different topics. Further research is required to explore HCPs’ knowledge about autism in general and, more specifically its effects on RSH. Exploration of HCPs’ capacity to provide satisfactory care in this domain and their willingness to do so could inform the development of resources to improve their capacity to address the RSH needs of autistic people AFAB. There may also be value in developing tools to support agency for autistic people wishing to initiate conversations about RSH with HCPs.

## Supplemental Material

sj-docx-1-aut-10.1177_13623613241290628 – Supplemental material for Unmet need for autism-aware care for gynaecological, menstrual and sexual wellbeing Supplemental material, sj-docx-1-aut-10.1177_13623613241290628 for Unmet need for autism-aware care for gynaecological, menstrual and sexual wellbeing by Richard O de Visser, Rachel Mosely, Julie Gamble-Turner, Laura Hull, Felicity Sedgewick, Charlotte Featherstone, Chella Quint OBE, Eloise Freeman and Marianna Karavidas in Autism
