# Supplementary material for: Unmet need for autism-aware care for gynaecological, menstrual and sexual wellbeing
Source: Autism. 2024 Oct 15;29(4):934–44. doi: 10.1177/13623613241290628 (PMC11967085; doi:10.1177/13623613241290628)
Supplement: sj-docx-1-aut-10.1177_13623613241290628 – Supplemental material for Unmet need for autism-aware care for gynaecological, menstrual and sexual wellbeing [file sj-docx-1-aut-10.1177_13623613241290628.docx]

**autism + sexual health care Qre**

**Q3** What is your age? [value]

**Q5** What sex were you assigned at birth?

Female (1)

Male (2)

Intersex or other (3)

**Q6** Is your gender identity the same as your sex assigned at birth?

Yes (1)

No, my gender identity is different to my sex assigned at birth (2) I’m not sure (3)

**Q7** How do you describe your gender? (you may tick more than one box) Transgender man (1)

Transgender woman (2)

Non-binary (3)

Genderqueer (4)

Genderfluid (5)

Gender expansive (6)

Pangender (7)

Agender (8)

Other (9)

**Q7_1** 1 = Autigender **Q7_2** 2 = non-binary **Q7_3** 3 = Agender **Q7_4** 4 = genderqueer **Q7_5** 5 = gendervague

**Q8** What is your ethnic background?

Asian / Asian British (1) Black / Black British (2) Middle Eastern (3)

Mixed heritage (4)

White / White British / Other White (5)

6 = Ashkenazi jewish

7 = Hispanic

**Q9** What is the highest level of education you have completed?

1. none of these
2. GCSE or equivalent
3. A level or equivalent
4. University degree or equivalent (e.g., BSc, BEd)
5. Higher degree (e.g., MA, PhD)
6. Other (please specify)

**Q10** Are you formally diagnosed as autistic?

1. Yes
2. No, but I am seeking or awaiting formal assessment
3. No, but I am self-diagnosed or identify as autistic

**Q11** How old were you when you were formally diagnosed as autistic? If you are unsure ...

**Q46** How old were you when you self-diagnosed as autistic or first identified as autistic?

**Q12** Have you ever been diagnosed with any other kind of neurodevelopmental condition ...?

Yes (1)

No (2)

**Q13** Please specify which other neurodevelopmental condition(s) you have been diagnosed with

[codes created based on participants’ responses] Q13_1 ADHD

Q13_2 dyslexia

Q13_3 dyspraxia

Q13_4 dyscalculia

Q13_5 epilepsy

Q13_6 Tourette's syndrome Q13_7 dysgraphia

**Q14** Do you have any ongoing conditions that affect your psychological wellbeing (e.g., depression, anxiety)?

Yes (1)

No (2)

**Q48** Please specify which condition(s) you have been diagnosed with [codes created based on participants’ responses]

Q48_1 anxiety / GAD Q48_2 depression Q48_3 eating disorder Q48_4 panic

Q48_5 OCD

Q48_6 PTSD

Q48_7 agoraphobia

Q48_8 personality disorder (borderline / unstable / dissociative) Q48_9 bipolar

Q48_10 body-focused repetitive disorder Q48_11 PMDD

Q48_12 functional neurological disorder Q48_13 long covid

**Q15** Do you have any ongoing conditions that affect your mobility ...? Yes (1)

No (2)

**Q49** Please specify which condition(s) you have been diagnosed with [codes created based on participants’ responses]

Q49_1 hypermobility Q49_2 fibromyalgia

Q49_3 ME/CFS

Q49_4 arthritis

Q49_5 Ehlers-Danlos Syndrome Q49_6 chronic pain

Q49_7 lipoedema

Q49_8 tachycardia

Q49_9 CHD

Q49_10 dysautonomia Q49_11 slipped disc / sciatica

Q49_12 femeroacetabular syndrome

Q49_13 slippping rib

**Q16** In general, how would you rate your overall physical health?

5 Excellent

1. Very good
2. Good
3. Fair
4. Poor

**Q17** In general, how would you rate your overall psychological wellbeing?

1. Excellent
2. Very good
3. Good
4. Fair
5. Poor

**Q18** How old were you when you had your first menstrual period? If you are unsure ... [value]

**Q19** Have you any experience of the menopausal transition? ...

1 No (1)

1. I am in perimenopause ...
2. I am post-menopause ...

88 Don't know (4)

**Q20** ... In relation to the regularity of your menstrual cycle, which of the statements below apply ...?

1. I currently have regular periods
2. I currently have irregular periods - but this is normal for me
3. I currently have irregular periods, or no periods, due to birth control, treatment...
4. I have irregular periods, or no periods, because of menopause

**Q21** ... Were your periods usually regular?

1. Yes
2. No

**Q23** How painful are your periods?

0 = not at all painful ... 10 = extremely painful '

**Q24** How painful were your periods?

0 = not at all painful ... 10 = extremely painful '

**Q26** How comfortable would you be discussing ... at your GP practice?

0 = not at all comfortable ... 10 = completely comfortable'

**q26_1 physical health**

**q26_2 psychological wellbeing q26_3 sexual health**

**q26_4 menstrual-related issues q26_5 menopause-related issues q26_6 autism**

**Q27** In healthcare consultations, who would you prefer to initiate conversations about ...

1. Me
2. Doctor
3. either / no preference
4. I would not want to discuss this

**q27_1 physical health**

**q27_2 psychological wellbeing q27_3 sexual health**

**q27_4 menstrual-related issues q27_5 menopause-related issues**

**Q28** ... how comfortable you would feel discussing your **physical health** ... at your GP practice.

0 = not at all comfortable ... 10 = completely comfortable

**q28_1** in-person consultation **q28_2** video consultation **q28_4** email consultation **q28_3** phone consultation

**Q29** ... how comfortable you would feel discussing your **psychological wellbeing** ...

0 = not at all comfortable ... 10 = completely comfortable

**q29_1** in-person consultation **q29_2** video consultation **q29_4** email consultation **q29_3** phone consultation

**Q30** ... how comfortable you would feel discussing your **sexual health** ...

0 = not at all comfortable ... 10 = completely comfortable

**q30_1** in-person consultation **q30_2** video consultation **q30_4** email consultation **q30_3** phone consultation

**Q31** ... how comfortable you would feel discussing **menstrual-related** issues ...

0 = not at all comfortable ... 10 = completely comfortable

**q31_1** in-person consultation **q31_2** video consultation **q31_4** email consultation **q31_3** phone consultation

**Q32** ... how comfortable you would feel discussing **menopause-related** issues ...

0 = not at all comfortable ... 10 = completely comfortable

**q32_1** in-person consultation **q32_2** video consultation **q32_4** email consultation **q32_3** phone consultation

**Q33** What would be your preferred method for discussing ... at your GP practice?

1. In-person
2. Video

4 email

1. phone

**q33_1** your physical health

**q33_2** your psychological wellbeing

**q33_3** your sexual health

**q33_4** menstrual-related issues

**q33_5** menopause-related issues

**Q34** Please add any comments to explain your answers above

**Q35** How often do doctors or nurses at your GP practice do each of the following?

1. Never
2. Sometimes
3. Usually
4. Always
5. not applicable

**q35_1** Explain things in a way that is easy to understand?

**q35_2** Listen carefully to you?

**q35_3** Provide printed versions of important information?

**q35_4** Accommodate your sensory needs?

**Q36** Please add any comments to explain your answers above

**Q37** ... how often do doctors or nurses at your GP practice do each of the following?

1. Never
2. Sometimes
3. Usually
4. Always
5. not applicable

**q37_1** Check that they are communicating with you in your preferred way ...

**q37_2** Give you enough time to process what they say? **q37_3** Check that you understand what they say? **q37_4** Give you the opportunity to ask questions?

**Q38** Please add any comments to explain your answers above

**Q39** How often do doctors or nurses at your GP practice seem to know each of the following?

1. Never
2. Sometimes
3. Usually
4. Always
5. not applicable

**q39_1** How your autism affects your sexual health?

**q39_2** How your autism affects your experience of menstruation?

**q39_3** How your autism affects your experience of menopause?

**Q40** Please add any comments to explain your answers above
